# The “full rectangle” sign: a novel method for ultrasonographic diagnosis of fetal aberrant right subclavian artery

**DOI:** 10.1007/s00404-024-07785-8

**Published:** 2024-11-05

**Authors:** Ettie Piura, Offra Engel, Neta Doctory, Ofer Markovitch

**Affiliations:** 1 Obstetrical & Gynecological Ultrasound Unit, Department of Obstetrics and Gynecology, Meir Medical Center, Kfar Saba, Israel; 2 School of Medicine, Faculty of Medical and Health Sciences, Tel Aviv University, Tel Aviv, Israel

**Keywords:** Right subclavian artery, Aberrant right subclavian artery, Prenatal ultrasound

## Abstract

**Objective:**

To evaluate the feasibility and accuracy of a novel ultrasonographic screening method for an aberrant right subclavian artery (ARSA) using the novel “full rectangle” method.

**Methods:**

This prospective study was conducted at a tertiary care center, September 2022 to February 2023. The study included unselected pregnant women at 14–38 weeks of gestation referred for routine or targeted anomaly scans. All participants underwent scanning by two experienced sonographers to ascertain the presence or absence of aberrant right subclavian artery (ARSA) using both conventional and novel “full rectangle sign” methods for validation purposes. This is a novel screening method for ARSA that demonstrates the retro-tracheal course at the level of the supra-aortic vessels, forming what we term the “full rectangle sign”.

**Results:**

A cohort of 138 patients was enrolled. The "full rectangle" sign was discerned in 6 fetuses with ARSA (4.3%), while the typical three-sided figure of the right subclavian artery was demonstrated in the remaining 132 fetuses (95.7%). The novel method demonstrated 100% feasibility and complete concordance with the conventional method.

**Conclusion:**

The study results indicate that the full rectangle sign serves as an effective and dependable screening tool for identifying ARSA. It offers the advantage of a clear, unobstructed view at a level unaffected by sternum shadowing and eliminates confusion with the azygous vein.

## What does this study add to the clinical work

**Table:** 

Routinely examining the three-sided figure vs. the full rectangle sign could enhance prenatal identification of aberrant right subclavian artery.	

## Introduction

The right subclavian artery (RSA) typically originates from the brachiocephalic artery (BCA), the first bifurcation of the aortic arch, and runs toward the right arm. However, in 1–2% of individuals [1, 2], the RSA arises aberrantly from the distal aortic arch as the fourth supra-aortic vessel, taking a retro-tracheal path toward the right arm. This condition, known as aberrant right subclavian artery (ARSA), has been observed in various genetic abnormalities, including Down syndrome [3], and has been associated with cardiac and extracardiac anomalies [4–11]. While ARSA is often asymptomatic, it may cause symptoms that include dysphagia, cough and stridor, resulting from compression of the trachea and esophagus caused by the aberrant anatomy of the aortic arch [12]. This study assessed the feasibility, reproducibility, and accuracy of a novel screening method for ARSA by demonstrating the retro-tracheal course at the level of the supra-aortic vessels, forming what we refer to as the "full rectangle sign”.

## Materials and methods

This prospective, cross-sectional study took place at a tertiary care center from September 2022 to February 2023. The study included pregnant women who were referred for a routine or targeted anomaly scan. Two sonographers, O.M. and E.P., with 20 and 9 years of scanning experience, respectively, performed the scans.

The primary objective was to determine the presence or absence of ARSA using two methods: the conventional method described by Chaoui et al., which is considered the “gold standard” as it has been validated in multiple studies and the novel "full rectangle" method. Intra-observer agreement and reproducibility of the diagnosis was evaluated by having the same sonographer assess the full rectangle twice during the scan. The inter-observer assessment was performed in a separate study that included 30 unselected patients who were scanned by two investigators (O.M and E.P.) in a blinded fashion. None of the patients were found to have a fetus with ARSA, using both methods. Both sonographers also reviewed the video clips of each ARSA case. There was full agreement between investigators. Furthermore, to ensure quality control and validation, the data analysis included only patients who were subsequently referred for echocardiography due to appropriate indications, such as pregestational maternal diabetes, cardiac or cardiac-associated fetal abnormalities, maternal administration of medication with teratogenic cardiac effects, and first-degree relative of the fetus with a congenital cardiac abnormality. A pediatric cardiologist examined and corroborated all ARSA and non-ARSA cases.

In the conventional approach, RSA visualization involves angling the transducer toward the right shoulder in an axial section at the three-vessel view level, to depict its position in the upper plane of the transverse aortic arch. When a fetus has an ARSA, it appears lower in the thorax compared to the typical right subclavian artery. In the three-vessel tracheal view, it manifests as a vessel coursing behind the trachea toward the right arm.

The full rectangle sign involves visualizing the aortic arch and supra-aortic vessels at the level of the fetal shoulders. Ideally, the fetus should be in a supine position. To demonstrate the full rectangle sign, the standard three-vessel trachea view must be obtained first. This view, described previously by Yagel et al., is achieved in an axial view of the chest and demonstrates the pulmonary artery, aorta, and the superior vena cava [13].

To visualize the supra-aortic vessels, the ultrasound transducer is then moved cranially to the fetal neck at the level of the shoulders. Understanding the spatial arrangement of the aortic arch and its branches in relation to neighboring structures greatly facilitates visualization of the three-sided figure and the full rectangle sign.

A cross-sectional view at this level reveals the ascending aorta on the right side, the left brachiocephalic vein taking a horizontal course, and the initial segment of the descending aorta on the left side, outlining a three-sided figure.

The ascending aorta stems from the left ventricle, located anteriorly and slightly to the right of the trachea. Moving across the midline, the aortic arch passes in front of the trachea and over the left main bronchus, proceeding toward the left side of the trachea. From this juncture, it descends alongside the thoracic esophagus. The fetal trachea displays a distinct ultrasound appearance, characterized by bright echogenic walls enclosing a sonolucent, fluid-filled lumen. This serves as a pivotal anatomical reference along the midline.

**Fig. 1:**
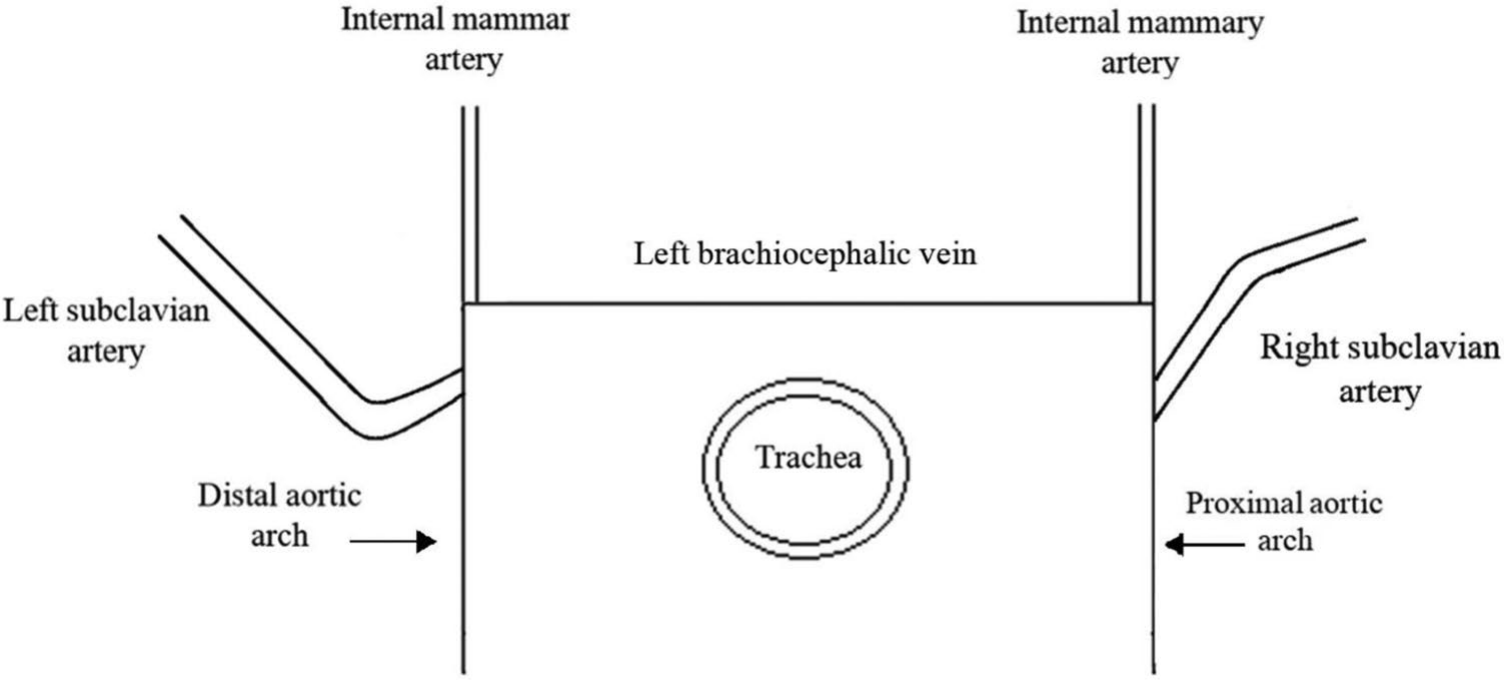
The “three-sided” sign. Schematic drawing of a transverse section at the level of the fetal shoulders, illustrating the course of the typical right subclavian artery (TRSA)

**Fig. 2:**
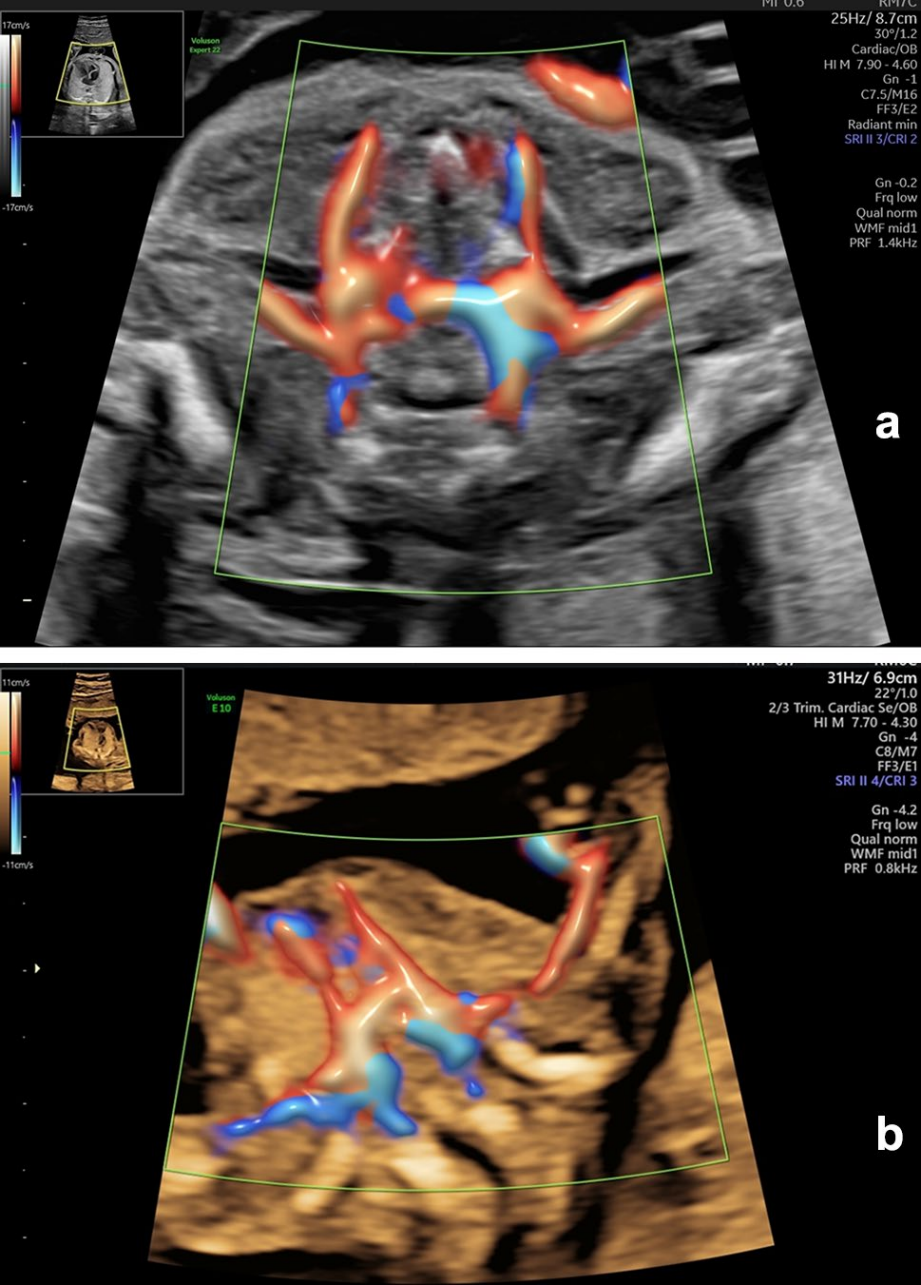
**a** Ultrasound image of the “three-sided” sign. A transverse section at the level of the fetal shoulders, illustrating the course of the typical right subclavian artery (TRSA) at 22 weeks of gestation. **b** Ultrasound image of the “three-sided” sign. A transverse section at the level of the fetal shoulders, illustrating the course of the typical right subclavian artery (TRSA) at 15 + 5 weeks of gestation

The typical right subclavian artery (TRSA) originates from the brachiocephalic trunk, located at the proximal right side of the horizontal aortic arch, which corresponds to the right upper corner of the three-sided figure. The left subclavian artery bifurcates from the distal left part of the horizontal aortic arch, corresponding to the left upper corner of the three-sided figure. The trachea is visible in the center of the section, and the cervical vertebrae can be observed posteriorly (Figs. 1, 2a, b).

By carefully examining the aortic segment at the level of the fetal shoulders, identification and tracking of the RSA origin become clear. This reveals its unique anatomical pathway: emerging as the first branch on the right side of the aortic arch in typical cases, or on the left distal aortic arch, in ARSA cases.

**Fig. 3:**
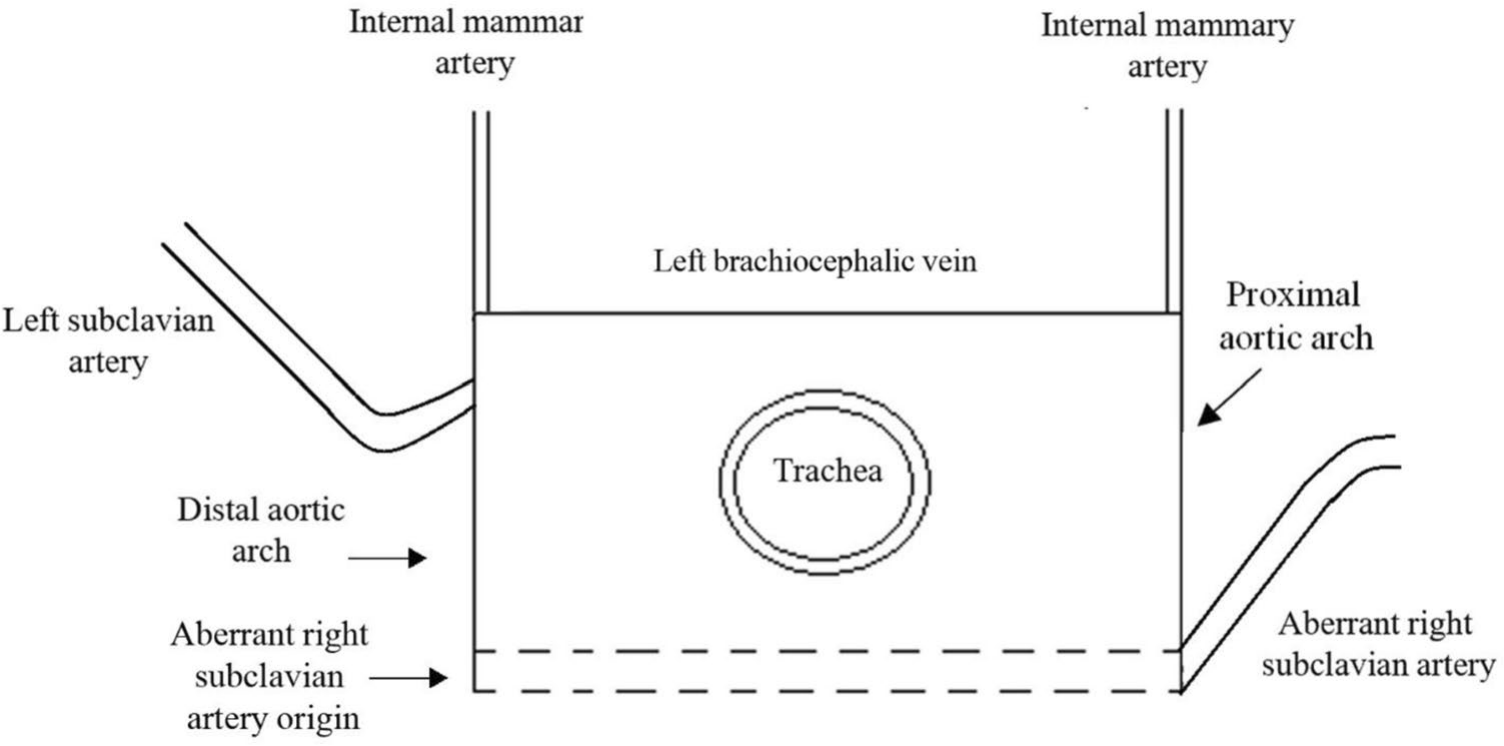
The “full rectangle” sign. Schematic drawing of a transverse section at the level of the fetal shoulders, illustrating the course of an aberrant right subclavian artery (ARSA). The ARSA originates from the distal aortic arch and travels behind the trachea toward the right arm

**Fig. 4:**
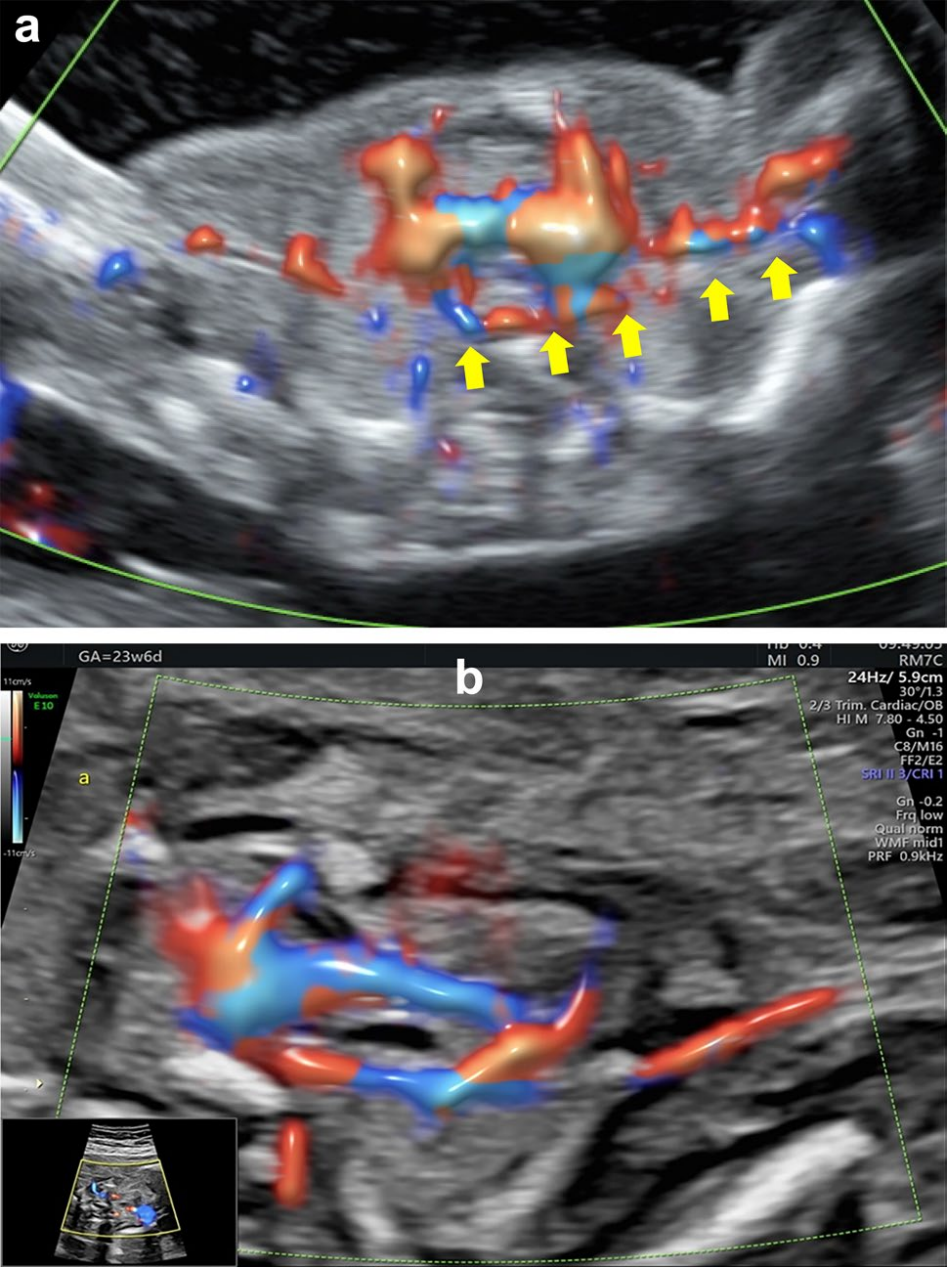
**a** Ultrasound image of the “full rectangle” sign. Transverse section at the level of the fetal shoulders, illustrating the course of the aberrant right subclavian artery (ARSA). (yellow arrows) at 15 weeks of gestation. **b** Ultrasound image of the “full rectangle” sign. Transverse section at the level of the fetal shoulders, illustrating the course of the aberrant right subclavian artery (ARSA; yellow arrows) at 23 + 6 weeks of gestation

In the case of an ARSA, the artery bifurcates from the distal aortic arch as the fourth supra-aortic vessel and takes a retro-tracheal course toward the right arm. When examining a transverse section at the level of the fetal shoulders, the retro-tracheal course of the ARSA adds a posterior line to the three-sided figure, resulting in the formation of the full rectangle sign. This sign fully surrounds the trachea, providing a visual indication of the presence of ARSA. Furthermore, the course of the ARSA from its origin at the descending aorta (representing the left lower corner of the full rectangle) as it traverses behind the trachea and extends into the right arm is clearly illustrated (Figs. 3, 4a, b).

The examinations were conducted using a Voluson E10 ultrasound machine (GE Healthcare, Milwaukee, WI, USA). A vaginal RIC 6–12 MHz probe was utilized for transvaginal scans at 12–15.6 weeks of gestation. Transabdominal scans with an abdominal RM6C 2–6 MHz convex probe were typically performed from 16 weeks of gestation and onward. Doppler was used, gradually lowering the pulse repetition frequency (PRF) to 0.8 Hz to detect the path of the right subclavian artery.

### Data analysis

Data regarding maternal demographics, medical history, fetal anomaly scan results, prenatal genetic evaluation, and postnatal outcomes were collected. The group with the typical three-sided figure formed by TRSA and the cases exhibiting the full rectangle sign formed by ARSA were compared. Maternal and fetal parameters were analyzed and compared between these two groups.

Categorial variables were summarized as frequency and continuous variables as median and interquartile range. Categorial variables were compared between those with and without ARSA using the Fisher exact test. The Mann–Whitney test was used for continuous variables.

The exact binominal method was used to calculate confidence intervals. All statistics were two sided and *p* < 0.05 was considered statistically significant. SPSS software (FBSCS statistics, Windows version 28) and NCSS 2022 statistical software (NCSS, LLC. Kaysville, UT, USA, ncss.com/software/ncss) were used for all data analyses.

## Results

A total of 138 unselected patients were enrolled in the study and scanned, either at a routine early (12–16 weeks; *n* = 20) anomaly scan, a routine mid-trimester (19–26 weeks; *n* = 29) anomaly scan, or during a targeted anomaly scan for various conditions and at different weeks of gestation (*n* = 89). We observed the three-sided figure of the TRSA in 132 fetuses (95.7%) and the full rectangle sign in 6 fetuses with ARSA (4.3%) at various gestational ages. In all these fetuses, ARSA was either ruled out or diagnosed using the conventional method, with no false positive cases. We correctly confirmed or ruled out the presence of the full rectangle sign in 100% of cases compared to the “gold standard” conventional ultrasonographic method [3]. In all cases, the presence of a three-sided figure or a full rectangle figure was identified twice during the same scan by the same investigator. There was complete agreement in all cases.

The medical history and pregnancy characteristics of the study group are presented in Table 1. We compared the TRSA group to the ARSA group. There were no statistically significant differences among the groups regarding maternal and pregnancy characteristics except for the mean pregnancy week at diagnosis, which was earlier for the ARSA cases (19.5 w vs. 26 w, *p* = 0.019; Table 1). All ARSA cases were isolated and subsequently referred for genetic counseling, where amniocentesis was recommended. Three of the six cases underwent amniocentesis, all with a normal microarray result. The latest diagnosis of ARSA was at 24 weeks. The three-sided figure and the full rectangle sign were also visible at term. To confirm that the distribution of the fetal weight percentiles in both groups followed a normal distribution (without increased incidence of small or large for gestational age infants), weight percentiles at the 25th, 50th and 75th of the study population were calculated. The close alignment of weight percentiles of both groups to the distribution of the study’s population percentiles, supports the conclusion of a normal distribution.

**Table 1:** Clinical variables

Variable*	3-sided figure sign—TRSA (*n* = 132)	Full rectangle sign—ARSA (*n* = 6)	*p* value
Singleton	113 (85.6%)	5 (83.3%)	
Twin pregnancy			0.616
DCDA twin pregnancy	12 (9.1%)	1 (16.7%)	
MCDA twin pregnancy	7 (5.3%)	0 (0%)	
Type of conception			0.629
Unassisted	122 (92.4%)	6 (100%)	
In vitro fertilization	10 (7.6%)	0 (0%)	
Fetal sex			0.162
Female	50 (37.9%)	4 (66.7%)	
Male	82 (62.1%)	2 (33.3%)	
Gestational age, weeks (mean)	26 ± 6.5	19.5 ± 4.4	0.019*
Maternal age, years (mean)	33.4 ± 5.6	30.9 ± 4.3	0.358
Gravidity (mean)	2.9 ± 2.0	3.3 ± 2.4	0.649
Parity (mean)	1.5 ± 1.5	1.5 ± 1.6	0.974
Fetal weight mean percentile	47.0 ± 29.5	53.2 ± 32.7	0.624
Mean fetal weight percentile distribution			
25th percentile	22.1	21.5	
50th percentile	49.1	59.3	
75th percentile	73.5	81.7	

Data are reported as mean standard deviation or *n* (%)

*TRSA* typical right subclavian artery, *ARSA* aberrant right subclavian artery, *DCDA* dichorionic diamniotic, *MCDA* monochorionic diamniotic

## Discussion

Prenatal detection of ARSA is very important due to its reported association with various genetic syndromes, particularly in non-isolated cases [3–14]. While a recent study found no association between ARSA and chromosomal abnormalities or major fetal malformations [14], other prenatal studies have reported a higher incidence of genetic abnormalities, particularly trisomy 21, and structural malformations in fetuses with ARSA [3–14].

Moreover, ARSA itself can occasionally lead to distressing symptoms by compressing the esophagus and trachea [15–19]. While large-scale cadaveric investigations have indicated a 1.23–2.2% prevalence of ARSA [1, 2], prenatal ultrasound studies involving unselected populations have reported a much lower prevalence of only 0.4% [17]. Similar findings of low ARSA prevalence have been observed in additional prenatal research [14, 18]. This discrepancy may be due to limited gestational weeks studied, which could exclude early ARSA cases that resulted in termination, or insufficient prenatal detection. When these studies were conducted, they all utilized the conventional method as the sole validated ARSA screening approach available. Notably, the lower prenatal prevalence of ARSA reported in comparison to pathological reports might stem from a potential underdiagnosis of this condition, prenatally.

The present study revealed a notably higher ARSA rate of 4.3%, surpassing rates reported in previous fetal studies [1, 2, 17, 18]. Importantly, our inclusion criteria focused on cases undergoing fetal echocardiography due to established cardiac risk factors. In contrast, 181 cases evaluated during the study period without referral to fetal echocardiography were excluded from the analysis. None of these cases were diagnosed with ARSA, indicating an overall prevalence of 2.2%, which aligns more closely with the prevalence figures from cadaveric investigations [1, 2].

All six ARSA cases identified in our study were isolated incidents. Among these, three underwent microarray testing and all results were normal. This trend aligns with earlier research indicating that isolated ARSA cases rarely exhibit chromosomal abnormalities [14, 19]. However, conflicting reports suggest a possible link between isolated ARSA and aneuploidy [4–10].

Relying on the conventional detection method is not always practical. An investigation evaluating its application during first and second trimester scans indicated a feasibility of 85.3% and 98%, respectively, culminating in an overall feasibility rate of 94% [19]. The researchers noted that feasibility seemed to be linked to factors such as fetal crown–rump length and maternal body mass index.

In contrast, our study achieved a 100% feasibility rate, utilizing a transvaginal approach until the 16th week of gestation and transitioning to a transabdominal approach beyond 16 weeks. Every instance showing a three-sided figure or a full rectangle sign was subsequently confirmed as normal or abnormal, when compared to the gold standard conventional method, without any false negative or false positive diagnoses. Furthermore, the intra-observer assessments validated the reproducibility and reliability of both the typical three-sided figure and the full rectangle sign in ARSA cases.

Kasif et al. proposed employing the “no ARSA” sign as a diagnostic marker for the normal right subclavian artery trajectory. In their research, they affirmed the regular emergence of the RSA by observing the bifurcation of the BCA at the three-vessel view level [20].

An additional ARSA screening method is valuable for two primary reasons. First, solely relying on the conventional approach, or another sign at the level of the three-vessel view, could result in overlooked ARSA cases due to the acoustic shadow caused by the sternum, clavicle, and rib cage, which obscures the retro-tracheal field at the level of the fetal chest. The full rectangle sign scanning method presented here offers significant advantages, including clear visualization above the level of the three-vessel view without shadowing from the sternum. This is because the ARSA originates from the right anterior mediastinal region and extends upward and posteriorly, passing behind the trachea at the lower level of the fetal neck, and ultimately reaching the right arm. Due to its more cranial level, the full rectangle sign is less affected by acoustic shadows. Additionally, positioning further away from the drainage of the azygous vein toward the superior vena cava at the three-vessel view level helps prevent confusion with the presence of an ARSA at the three-vessel view level. This positioning also offers a thorough illustration of the origin of the ARSA from the descending aorta, tracking its pathway behind the trachea toward the right arm.

Based on our research, this study proposes an approach for diagnosing the course of an ARSA based on demonstrating the anatomy of the vessels. Introducing an additional confirmatory screening method for ARSA enhances our ability to identify cases where there is uncertainty regarding whether the failure to demonstrate the typical right subclavian course is genuinely accurate or an oversight.

In the context of scanning a typically left-oriented aortic arch, it is prudent to consider potential variants. Many aortic arch anomalies can be understood through the double arch theory outlined by Edwards [21, 22]. The occurrence of ARSA stems from improper regression of the left aortic arch segment lying between the right subclavian and right carotid arteries. Additional variations of the left aortic arch include distinct origins of common carotid arteries, subclavian arteries, and vertebral arteries directly from the arch. There is also the possibility of a shared trunk giving rise to the right BCA and left common carotid artery, or an independent emergence of the left vertebral artery proximal to the left subclavian artery [23]. In these instances, an abnormal number of vessels arise either directly from the aortic arch or from its primary arising vessel.

Thus, if an artery to the right arm arises from the upper right corner of the three-sided figure without an artery completing the shape by following a retro-tracheal path, the scanner can exclude the possibility of an unnoticed ARSA due to an arch variant. This is particularly crucial when dealing with non-isolated cases.

The simplicity and ease of use of the full rectangle sign method make it highly practical. By integrating this straightforward technique into routine scanning of low- and high-risk populations, we could potentially enhance the prenatal detection rate of ARSA and subsequently refine prenatal counseling.

The aim of our study was to introduce a novel, simple, and feasible method for the prenatal ultrasound diagnosis of ARSA. This method can be used in conjunction with, or as an alternative to the conventional approach at the three-vessel view level. Demonstrating the statistically significant superiority of one method over the other was beyond the scope of this study. A future study comparing the diagnostic accuracy of each method individually is necessary to address this question.

The present study boasts several strengths, including its prospective design. By including only cases confirmed through fetal echocardiography, it effectively mitigates the risk of false diagnoses regarding the course of the right subclavian artery. Moreover, the ease of generating the transverse upper section of the neck–shoulder level, and the direct evaluation of the retro-tracheal space for aberrant passage of the right subclavian artery at the broad gestational age range covered in our study, enhances its internal validity across all three trimesters.

It is important to acknowledge the study’s limitations. The choice of a tertiary reference center may contribute to the high prevalence of ARSA observed, which might have been influenced by referrals for targeted scans due to diverse fetal anomalies. In this regard, we took precautions by refraining from using statistical tests influenced by disease prevalence, such as positive and negative predictive values. In addition, the heightened feasibility rate might be attributed to the advanced expertise of sonographers in a tertiary care setting.

## Conclusions

This study introduces an innovative, uncomplicated, and viable screening approach for ARSA termed the “full rectangle sign”. This method demonstrates commendable performance when contrasted with the conventional technique. The outcomes of our study suggest it is a feasible, reproducible, and dependable screening tool for ARSA.

Routinely incorporating the examination of the three-sided figure vs. the full rectangle sign could potentially enhance the prenatal identification of ARSA cases.

## Data Availability

The data supporting the findings of this study can be obtained from the corresponding author (E.P.), upon request. These data are not publicly accessible because they contain information that could compromise the privacy of the research participants.

## References

[1] Natsis K, Didagelos M, Gkiouliava A et al (2017) The aberrant right subclavian artery: cadaveric study and literature review. Surg Radiol Anat 39:559–565. 10.1007/s00276-016-1796-5 27999944 10.1007/s00276-016-1796-5

[2] Polednak AP (2017) Prevalence of the aberrant right subclavian artery reported in a published systematic review of cadaveric studies: the impact of an outlier. Clin Anat 30:1024–1028. 10.1002/ca.22905 28514512 10.1002/ca.22905

[3] Chaoui R, Heling K-S, Sarioglu N et al (2005) Aberrant right subclavian artery as a new cardiac sign in second- and third-trimester fetuses with down syndrome. Am J Obstet Gynecol 192:257–263. 10.1016/j.ajog.2004.06.080 15672034 10.1016/j.ajog.2004.06.080

[4] Esmer AC, Gul A, Nehir A et al (2013) Detection rate of trisomy 21 in fetuses with isolated and non-isolated aberrant right subclavian artery. Fetal Diagn Ther 34:140–145. 10.1159/000354650 24051543 10.1159/000354650

[5] Paladini D, Sglavo G, Pastore G et al (2012) Aberrant right subclavian artery: incidence and correlation with other markers of down syndrome in second-trimester fetuses. Ultrasound Obstet Gynecol 39:191–195. 10.1002/uog.10053 21793087 10.1002/uog.10053

[6] Gul A, Corbacioglu A, Bakirci IT, Ceylan Y (2012) Associated anomalies and outcome of fetal aberrant right subclavian artery. Arch Gynecol Obstet 285:27–30. 10.1007/s00404-011-1907-9 21487731 10.1007/s00404-011-1907-9

[7] Fehmi Yazıcıoğlu H, Sevket O, Akın H et al (2013) Aberrant right subclavian artery in down syndrome fetuses. Prenat Diagn 33:209–213. 10.1002/pd.4042 23319208 10.1002/pd.4042

[8] Zalel Y, Achiron R, Yagel S, Kivilevitch Z (2008) Fetal aberrant right subclavian artery in normal and down syndrome fetuses. Ultrasound Obstet Gynecol 31:25–29. 10.1002/uog.5230 18098348 10.1002/uog.5230

[9] Borenstein M, Cavoretto P, Allan L et al (2008) Aberrant right subclavian artery at 11 + 0 to 13 + 6 weeks of gestation in chromosomally normal and abnormal fetuses. Ultrasound Obstet Gynecol 31:20–24. 10.1002/uog.5226 18157795 10.1002/uog.5226

[10] Borenstein M, Minekawa R, Zidere V et al (2010) Aberrant right subclavian artery at 16 to 23 + 6 weeks of gestation: a marker for chromosomal abnormality. Ultrasound Obstet Gynecol 36:548–552. 10.1002/uog.7683 20503237 10.1002/uog.7683

[11] Scala C, Leone Roberti Maggiore U, Candiani M et al (2015) Aberrant right subclavian artery in fetuses with down syndrome: a systematic review and meta-analysis. Ultrasound Obstet Gynecol 46:266–276. 10.1002/uog.14774 25586729 10.1002/uog.14774

[12] Cai M et al (2022) Fetal aberrant right subclavian artery: associated anomalies, genetic etiology, and postnatal outcomes in a retrospective cohort study. Front Pediatr 10:895562. 10.3389/fped.2022.895562 35722491 10.3389/fped.2022.895562 PMC9203729

[13] Yagel S, Arbel R, Anteby EY, Raveh D, Achiron R (2002) The three vessels and trachea view (3VT) in fetal cardiac scanning. Ultrasound Obstet Gynecol 20(4):340–345. 10.1046/j.1469-0705.2002.00801.x 12383314 10.1046/j.1469-0705.2002.00801.x

[14] Kaya M (2024) Postnatal outcome of fetal aberrant right subclavian artery: a single center study. Arch Gynecol Obstet 310:129–133. 10.1007/s00404-024-07488-0 38555333 10.1007/s00404-024-07488-0

[15] Hanneman K, Newman B, Chan F (2017) Congenital variants and anomalies of the aortic arch. Radiographics 37:32–51. 10.1148/rg.2017160033 27860551 10.1148/rg.2017160033

[16] Freed K, Low VH (1997) The aberrant subclavian artery. AJR Am J Roentgenol 168:481–484. 10.2214/ajr.168.2.9016231 9016231 10.2214/ajr.168.2.9016231

[17] Song MJ, Han BH, Kim YH et al (2017) Prenatal diagnosis of aberrant right subclavian artery in an unselected population. Ultrasonography 36:278–283 28322033 10.14366/usg.16046 PMC5494869

[18] Gursoy Erzincan S, Karamustafaoglu Balci B, Tokgoz C, Kalelioglu IH (2017) Incidence of an aberrant right subclavian artery on second-trimester sonography in an unselected population. J Ultrasound Med 36:1015–1019. 10.7863/ultra.16.05075 28258603 10.7863/ultra.16.05075

[19] Rembouskos G, Passamonti U, De Robertis V et al (2012) Aberrant right subclavian artery (ARSA) in unselected population at first and second trimester ultrasonography. Prenat Diagn 32:968–975. 10.1002/pd.3942 22847746 10.1002/pd.3942

[20] Kassif E, Tsur A, Shust-Barequet S et al (2020) The “no ARSA” sign: A novel method of prenatal screening for aberrant right subclavian artery. J Clin Med. 10.3390/jcm9082658 32824459 10.3390/jcm9082658 PMC7463697

[21] Edwards JE (1953) Malformations of the aortic arch system manifested as vascular rings. Lab Invest 2:56–75 13036024

[22] Edwards JE (1948) Anomalies of the derivatives of the aortic arch system. Med Clin North Am 32:925–949. 10.1016/s0025-7125(16)35662-0 18877614 10.1016/s0025-7125(16)35662-0

[23] Kellenberger CJ (2010) Aortic arch malformations. Pediatr Radiol 40:876–884. 10.1007/s00247-010-1607-9 20354848 10.1007/s00247-010-1607-9

